# Age-associated changes in 4D flow CMR derived Tricuspid Valvular Flow and Right Ventricular Blood Flow Kinetic Energy

**DOI:** 10.1038/s41598-020-66958-y

**Published:** 2020-06-18

**Authors:** Natasha Barker, Hamza Zafar, Benjamin Fidock, Alaa Elhawaz, Abdallah Al-Mohammad, Alexander Rothman, David G. Kiely, R. J. van der Geest, Jos Westenberg, Andrew J. Swift, James M. Wild, Sven Plein, Pankaj Garg

**Affiliations:** 1 Department of Infection, Immunity & Cardiovascular Disease, University of Sheffield, Sheffield, UK; 2 Sheffield Teaching Hospitals NHS Foundation Trust, Sheffield, UK; 3 Insigneo Institute for in-silico Medicine, Sheffield, UK; 4 Cardiovascular Imaging Group, Department of Radiology, Leiden University Medical Centre, Sheffield, UK; 5 Division of Biomedical Imaging, Leeds Institute of Cardiovascular and Metabolic Medicine, University of Leeds, Leeds, UK

**Keywords:** Cardiology, Medical research

## Abstract

Assessment of right ventricular (RV) diastolic function is not routinely carried out. This is due to standard two-dimensional imaging techniques being unreliable. Four-dimensional flow (4D flow) derived right ventricular blood flow kinetic energy assessment could circumvent the issues of the current imaging modalities. It also remains unknown whether there is an association between right ventricular blood flow kinetic energy (KE) and healthy ageing. We hypothesise that healthy ageing requires maintaining normal RV intra-cavity blood flow as quantified using KE method. The main objective of this study was to investigate the effect of healthy ageing on tricuspid through-plane flow and right ventricular blood flow kinetic energy. In this study, fifty-three healthy participants received a 4D flow cardiovascular magnetic resonance (CMR) scan on 1.5 T Philips Ingenia. Cine segmentation and 4D flow analysis were performed using dedicated software. Standard statistical methods were carried out to investigate the associations. Both RV E-wave KEi_EDV_ (r = −0.3, P = 0.04) and A-wave KEi_EDV_ (r = 0.42, P < 0.01) showed an association with healthy ageing. Additionally, the right ventricular blood flow KEi_EDV_ E/A ratio demonstrated the strongest association with healthy ageing (r = −0.53, P < 0.01) when compared to all RV functional and haemodynamic parameters. Furthermore, in a multivariate regression model, KEi_EDV_ E/A ratio and 4D flow derived tricuspid valve stroke volume demonstrated independent association to healthy ageing (beta −0.02 and 0.68 respectively, P < 0.01). Ageing is independently associated with 4D flow derived tricuspid stroke volume and RV blood flow KE E/A ratio. These novel 4D flow CMR derived imaging markers have future potential for RV diastolic assessment.

## Introduction

Age is an independent risk factor for the development of heart failure(HF), and acute heart failure is the commonest presentation in the elderly^1,2^. With ageing, the heart undergoes structural and functional changes. With ageing, the heart adapts to vascular stiffening associated with increased thickness of the left ventricular wall and fibrosis. These changes, in turn, leads to diastolic failure secondary to increased afterload. Additionally, pulmonary vasculature is likely to get affected by age-associated arterial remodelling, resulting in the stiffness of pulmonary vasculature thus leading to elevated PAPs. The ability of the heart to adapt to physiological changes and compensatory mechanisms also diminishes with ageing including changes in contractility, maximal heart rate, end-systolic and diastolic volumes and increased pulse pressure with elevated left heart filling pressures due to blood vessel stiffening. These changes lower the threshold for the development of hypertension, diastolic dysfunction and heart failure^3,4^. The left ventricular (LV) functional and structural changes associated with ageing are well established and are in routine use for the assessment of systolic and diastolic LV function^5^. The LV ageing process can potentially be determined by mitral inflow derived flow velocities, and, as more recently demonstrated, LV blood flow kinetic energy (KE)^6^.

Interestingly, only a few studies have described the impact of ageing on right ventricular (RV) structure and function^7,8^. Similar to the LV, tricuspid valve (TV) inflow and RV blood flow may be better associated with ageing than the usual functional parameters and thus could play a role in the RV diastolic functional assessment which is not routinely done. The studies have also shown the impact of ageing on physiological changes in left and right ventricular ejection fraction (EF), end-systolic volume (ESV) and end-diastolic volume (EDV) in cohort with normal cardiac functional and structural values. Hence for identification of abnormality and prediction of patient outcome, it is prudent to understand the age-related normal structural and physiological cardiac functions^9^. These findings underline the importance of using age-adapted values as the standard of reference when evaluating CMR studies.

RV diastolic dysfunction (RVDD) has been defined as an increase in RV filling pressures, caused by passive (RV chamber stiffness) and active (impaired RV relaxation) mechanical abnormalities of the ventricular muscle function during diastole^10^. RVDD is an important prognostic marker in several cardiopulmonary disorders; such as - pulmonary embolism, pulmonary arterial hypertension and congestive heart failure^11–16^. The changes in RV function, with age, are mainly due to diastolic filling mechanisms, however there is no direct impact on RV systolic function. This was demonstrated by Lindqvist *et al*. where they used tissue Doppler methods to define RV function and conventional Doppler to study RV filling properties^17^. They demonstrated that age does not affect systolic RV function. The changes in RV function due to age are related to the diastolic filling velocities, which mirror those of the LV. In their study, the tricuspid E/A ratio was conversely associated with ageing. Even though RVDD can be assessed by conventional imaging methods, we still need semi-automated robust methods which reduce operator variability and can be used as reliable imaging biomarkers.

We hypothesise that the TV flow quantified by the advanced retrospective valve tracking (RVT) method and RV blood flow quantified by novel kinetic energy (KE) mapping using four-dimensional flow (4D flow) cardiovascular magnetic resonance (CMR) will demonstrate stronger association to ageing when compared with standard RV functional parameters. Hence, the main aim of this study was to investigate the association of RVT quantified tricuspid flow and RV blood flow KE parameters to age. Secondly, we aimed to establish the reproducibility of the novel RV KE parameters for clinical translation.

## Methods

### Study population

Healthy volunteers between the ages of 20 to 80 year, were prospectively recruited from two centres: Leeds, UK and Leiden, the Netherlands (32 males; mean age, 41.5 ± 17; range 20–73; and 21 females; mean age, 50.1 ± 16.8; range 27–80), as previously published^5^. Patients had no history or symptoms of cardiovascular disease, they were not on cardiovascular medications and had no contraindications to CMR. The study protocol was approved by the National Research Ethics Service (12/YH/0169) in the UK and the institutional Medical Ethical Committee (P11.136) in Leiden. The study complied with the Declaration of Helsinki and all healthy volunteers gave written informed consent.

### CMR protocol and image acquisition

CMR was performed on a dedicated cardiovascular 1.5Tesla Philips Ingenia system equipped with a 28-channel coil and Philips dStream digital broadband MR architecture technology. The CMR protocol has been described before^6^. The CMR protocol included the following: cine imaging including short-axis contiguous stack. All cines were acquired with a balanced steady-state free precession (bSSFP), single-slice breath-hold sequence. Typical parameters for bSSFP cine were as follows: SENSE factor 2, flip angle 60°, echo time (TE) 1.5 milliseconds, repetition time (TR) 3 milliseconds, field of view 320–420 mm according to patient size, slice thickness 8 mm, and 30 phases per cardiac cycle.

For whole heart 4D flow, the field of view (FOV) was planned in trans-axial plane making sure the whole heart was in FOV. If the necessary number of slices was increased. 4D flow was done using fast field echo (FFE) pulse sequence (EPI based, 3D) with retrospective ECG-triggering. The acquisition voxel size was kept as close as possible to 3 × 3 × 3 mm3. Field-of-view and number of slices (i.e., the 3D volume) were adapted to the subject’s size. The standard scan parameters were: echo time 3.5 ms, repetition time 10 ms, flip-angle 10°, field-of-view 400 mm, number of signal averages 1. VENC 150 cm/sec. Acceleration was achieved by Echo Planar Imaging with EPI factor 5. Free-breathing was allowed, and no respiratory motion compensation was used^6^. The number of slices was 40 with a temporal resolution of 40 ms. This sequence has been comprehensively validated before for flow quantification^18,19^. The number of reconstructed phases was set to 30. The 4D flow encoding was performed by standard 4-point encoding.

### 4D flow error corrections and quality checks

Online/offline 4D flow data quality assurance checks, Maxwell correction, phase unwrapping and spatial misalignment of 4D flow data were done as already described in a previous publication from our group^6^. The effects of concomitant gradient terms were compensated by Maxwell correction methods. Remaining background errors were corrected by the local phase correction (LPC) filter on the CMR scanner performed in a two-dimensional way - slice by slice^6^. The LPC is a magnitude-weighted spatial low pass filter; pixels that are expected to be part of the static background are used with a higher weight than noisy background pixels or pixels that are expected to contain flow to determine the local phase offset. LPC uses surrounding tissue to determine “static” areas.

All 3D phase-contrast data sets were investigated for phase aliasing artefacts. If present then phase unwrapping was performed as per previously published guidelines on phase-contrast methods^20^. Additionally, any spatial misalignment of 4D flow data to cine imaging was corrected before any flow analysis was performed. This was done by visualising streamlines in 4-chamber view at peak systole and repositioning them over descending aorta. Similar checks were done during diastole in 4-chamber and 2-chamber views for peak mitral inflow streamlines.

### Image analysis

Image analysis was undertaken at Sheffield and Leiden. Health control data from Leiden was analysed only at Leiden. All study images were analysed by PG (>5 years’ experience in advanced CMR techniques), NB (1-year experience in advanced CMR techniques), BF (1-year experience in advanced CMR techniques, BF carried out the blinded interobserver reliability tests) and RVDG (>5 years’ experience in advanced CMR techniques). Images were evaluated offline using research software (MASS; Version 2019EXP, Leiden University Medical Centre, Leiden, The Netherlands). RV volumes and ejection fraction were both devised by standard methods and these are outlined below (Møgelvang *et al*., 1988). Tricuspid annular plane systolic excursion (TAPSE) was determined by adapting the previous methodology used for mitral annular plane systolic excursion^21^. Right atrial area (RAA) was contoured in the four-chamber view just prior to the opening of the tricuspid valve (end-systolic phase).

### Valve tracking based TV flow quantification

All two-dimensional (2D) tricuspid inflow assessments were performed using validated techniques including retrospective valve tracking, with measurement planes positioned perpendicular to the inflow direction on two- and four-chamber cines^22,23^. Background velocity correction (i.e., for correction of through-plane motion and phase offset) was derived from the velocity sampled in the myocardium in the reformatted dynamic phase contrast plane. Contour segmentation was performed manually. Tricuspid inflow metrics computed included: tricuspid valve stroke volume (ml), peak early tricuspid inflow velocity (E-wave velocity), peak late tricuspid inflow velocity (A-wave velocity) and E/A ratio respectively.

### 4D flow RV KE mapping

The LV short-axis segmentation was previously drawn by RVDG^6^. The RV segmentation was done manually in phases one, six, twelve, eighteen and twenty-four in each short-axis slice. The software track system was then used to automatically propagate time-resolved contours to the intermediate phases^6^. Quality check for each generated contour was performed visually for any possible projection errors and manually corrected whenever needed. As the RV endocardial contours are the key determinants of blood flow within the RV, a detailed stepwise methodology and quality checks are further described in the online Supplementary Document.

**Figure 1:**
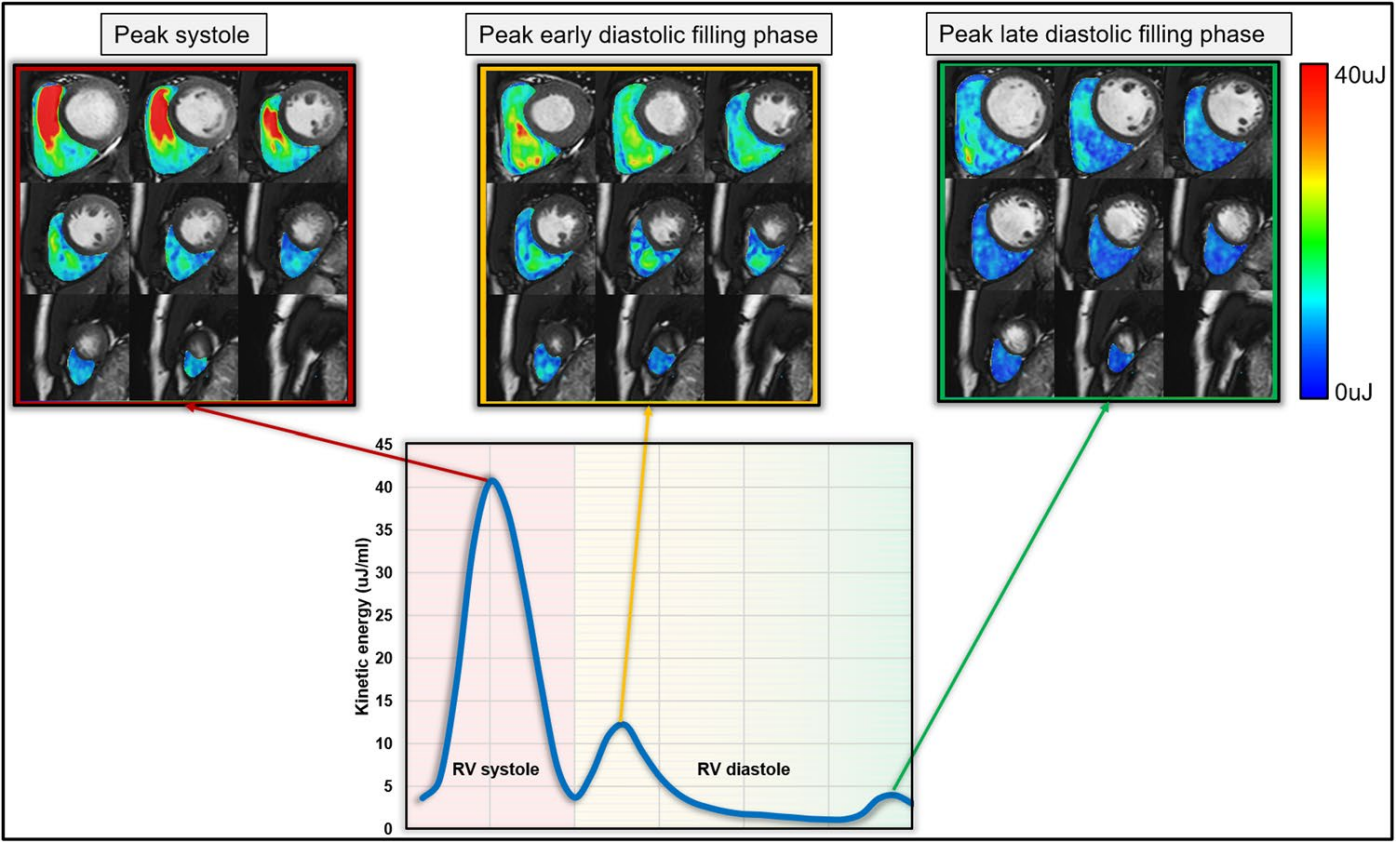
Overview of kinetic energy co-registered maps on short-axis cine.

For calculation of RV blood flow KE parameters, the segmentation for RV functional analysis was used to compute a three-dimensional RV volume similar to our pervious work^6^. For the 3D RV volume, the RV radius for a given angle and RV level was derived by linear interpolation^6^. This time-resolved, RV volume was constructed by representing the mesh in cylindrical coordinates^6^. Finally, this time-resolved RV mesh was applied to the raw velocity-encoded data as previously described^24^. Correction for translational and rotational misalignment between the short-axis cine and the 4D Flow CMR acquisition was performed using automated image registration as previously described^25^. For each volumetric element (voxel) the KE was computed using the following formula:

$$KE=1/2{\rho }_{blood}\cdot {V}_{voxel}\cdot {v}_{voxel}2$$

with ρ_blood_ being the density of blood (1.06 g/cm^3^), V_voxel_ the voxel volume and v_voxel_ the velocity magnitude of the corresponding voxel^6^. For each cardiac phase, the total KE within the RV was obtained by summation of the KE of every voxel. All KE parameters were normalized to the RV end-diastolic volume (KEi_EDV_) and accordingly reported in μJ/ml^6^. Time-resolved KE curves were generated to derive physiologically relevant parameters, including: global RV KEi_EDV_ (the mean KE of RV blood flow throughout the entire cardiac cycle), systolic KEi_EDV_ (the KE of the RV blood flow during systole), diastolic KEi_EDV_ (the KE of the RV blood flow during diastole), peak E-wave KEi_EDV_ (the peak KE of the RV blood flow during early tricuspid filling), peak A-wave KEi_EDV_ (the peak KE of the RV blood flow during late tricuspid filling) and KEi_EDV_ E/A ratio (the ratio of RV peak E-wave KE to RV peak A-wave KE) (Fig. 1)^6^.

### Intra-/inter-observer reproducibility

For interobserver reproducibility, NB and BF contoured the short-axis RV cine volumetric stack in 20 random study subjects and both were blinded to each other’s analysis. For intraobserver reproducibility, NB re-analysed the RV short-axis cines for the same 20 subjects after 2 months. The automated KE parameters were generated both for Intra and interobserver reproducibility through new endocardial contouring respectively.

### Statistical analysis

Statistical analysis was performed using IBM SPSS Statistics 21.0 and on MedCalc (version: 19.0.5)^6,26^. Continuous measurements are presented as mean ± standard deviation^6^. Demographic comparisons were performed with an independent samples t-test. Intra-/inter-observer reliability tests were done by the coefficient of variability. In different quartiles of age group, the Kruskal-Wallis H test was used to perform Dunn posthoc analysis. Association of age to KE parameters was carried out by Spearman’s rank correlation coefficient test. In multivariate analysis, a forward-conditional method was used for regression and parameters with statistical significance from one-way analysis (p  <  0.05) were chosen for multivariate analysis. A p-value < 0.05 was considered statistically significant.

## Results

### Data summary

All 53 healthy volunteers completed the full study protocol. The research cohort contained 21% females with a mean age of 50 ± 17 years and 32 males with a mean age of 42 ± 17. Table 1 demonstrates a full summary of the study cohorts’ demographic characteristics.

**Table 1:** Demographic characteristics, kinetic energy and haemodynamic variables of the 53 healthy volunteers involved in this research project. These values have been separated into entire study cohort, male and female. The data are presented as mean ± standard deviation.

	Healthy Volunteers	Female (N = 21)	Males (N = 32)	P-value
Age (years)	45 ± 17	50 ± 17	42 ± 17	0.08
Body surface area (m^2^)	1.85 ± 0.2	1.7 ± 0.2	1.94 ± 0.13	<0.01
RV volumetric and functional assessment				
RVEDVi (ml/m^2^)	84.77 ± 23.1	81.31 ± 21.2	87.03 ± 24.3	0.38
RVESVi (ml/m^2^)	35.74 ± 11.7	33.67 ± 8.95	37.09 ± 13.2	0.3
RV SVi (ml/m^2^)	51.19 ± 10.1	50.78 ± 10.2	51.46 ± 10.3	0.81
RV EF (%)	59.46 ± 6.14	60.39 ± 5.02	58.86 ± 6.78	0.38
Right atrial area (mm)	23.04 ± 4.09	22.31 ± 3.05	23.53 ± 4.64	0.3
RAAi (mm)	12.54 ± 2.21	13.14 ± 2.08	12.14 ± 2.23	0.11
TAPSE	28.36 ± 6.47	30.12 ± 7.34	27.2 ± 5.66	0.11
Retrospective valve tracking assessment of tricuspid diastolic inflow				
RV peak E-wave velocity cm/s	49.85 ± 10.1	49.69 ± 8.13	49.95 ± 11.3	0.93
RV peak A-wave velocity cm/s	38.8 ± 10.9	38.97 ± 9.2	38.68 ± 12.1	0.93
RV KE E/A ratio	1.37 ± 0.4	1.33 ± 0.31	1.4 ± 0.45	0.54
TV SV (ml)	87.9 ± 23	81.4 ± 18	92.1 ± 25	0.1
RV KE assessment				
RV global KEi_EDV_ µJ/ml	4.6 ± 1.89	4.57 ± 1.07	4.63 ± 2.29	0.92
RV minimum KEI_EDV_ µJ/ml	0.71 ± 0.57	0.84 ± 0.8	0.63 ± 0.34	0.19
RV systolic KEi_EDV_ µJ/ml	8.12 ± 3.88	7.4 ± 1.89	8.6 ± 4.72	0.27
RV diastolic KEi_EDV_ µJ/ml	2.68 ± 1.08	2.89 ± 0.96	2.53 ± 1.14	0.24
RV peak E-wave KEi_EDV_ µJ/ml	5.53 ± 2.81	5.73 ± 2.94	5.4 ± 2.76	0.67
RV peak A-wave KEi_EDV_ µJ/ml	4.59 ± 1.88	5.15 ± 1.66	4.22 ± 1.94	0.08
RV KEi_EDV_ E/A ratio	1.51 ± 0.84	1.29 ± 0.57	1.65 ± 0.97	0.14

RV = right ventricle, RVEDVi = right ventricular end-diastolic volume indexed, RVESVi = right ventricular end-systolic volume indexed. RV SV = RV stroke volume indexed RV EF = RV ejection fraction, RAAi = right atrial area indexed, TAPSE = tricuspid annular plane systolic excursion, KEiEDV = kinetic energy indexed to end-diastolic volume. The P-value shows the statistical difference of the characteristics between the sexes.

### Study population

Table 1 shows no significant sex differences for any of the indexed CMR data. Furthermore, there was no significant difference for RV peak-E and peak-A wave velocities. Global RV KEi_EDV_ was 4.6 ± 1.89 µJ/ml (mean ± SD), with an RV systolic KEi_EDV_ of 8.12 ± 3.88 µJ/ml and mean RV diastolic KEi_EDV_ 2.68 ± 1.08 µJ/ml. Additionally, peak E-wave KEi_EDV_ was 5.53 ± 2.81 µJ/ml and peak A-wave KEi_EDV_ 4.59 ± 1.88 µJ/ml. This resulted in an average KEi_EDV_ E/A ratio of 1.51 ± 0.84 respectively.

### Sex differences

There were no significant sex differences for global RV KEi_EDV_ (4.57 ± 1.07 vs 4.63 ± 2.29, P = 0.92) assessment. This was also the case for RV systolic KEi_EDV_ (P = 0.27) and diastolic KEi_EDV_ (P = 0.24) assessments. Males exhibited a higher RV peak A-wave KEi_EDV_ (5.15 ± 1.66 vs 4.22 ± 1.94), although this did not reach statistical significance (P = 0.08). There was no difference in RV peak E-wave KEi_EDV_ (P = 0.67) and therefore, the KEi_EDV_ E/A ratio was very similar (1.29 ± 0.57 vs 1.65 ± 0.97, P = 0.14).

### Age-associated RV functional changes

Indexed RV end-diastolic volume (RVEDVi), end-systolic volume (RVESVi) and stroke volume (RVSVi) demonstrated a negative association with advancing age (P < 0.01) (Table 2). However, RV ejection fraction (RV EF) and indexed right atrial area (RAAi) had no significant association with age. TAPSE demonstrated a negative modest association with age (R = −0.307, P = 0.03).

**Table 2:** This table demonstrates the associations between age and RV functional parameters, as well as retrospective valve tracking, derived tricuspid valve inflow parameters and right ventricular blood flow kinetic energy parameters. The final section of the table displays the association between two dimensional and 4D right ventricular blood flow parameters.

		Spearman’s rank correlation coefficient	P-value
RV functional parameters	RVEDVi	−0.485	<0.01
	RVESVi	−0.478	<0.01
	RV SVi	−0.436	<0.01
	RV EF (%)	0.261	0.06
	RAAi	0.031	0.83
	TAPSE	−0.307	0.03
RVT derived TV inflow parameters	Peak E-wave velocity	−0.165	0.24
	Peak A-wave velocity	0.4	<0.01
	E/A ratio	−0.458	<0.01
	TV SV	−0.434	<0.01
RV blood flow KE parameters	RV global KEi_EDV_	−0.074	0.60
	RV systolic KEi_EDV_	−0.106	0.45
	RV diastolic KEi_EDV_	−0.172	0.22
	RV peak E-wave KEi_EDV_	−0.3	0.04
	RV A-wave KEi_EDV_	0.42	<0.01
	KEi_EDV_ E/A ratio	−0.531	<0.01
Association of 4D diastolic RV KE parameters to 2D tricuspid inflow parameters			
RV peak E-wave velocity to RV peak E-wave KEi_EDV_		0.44	<0.01
RV Peak A-wave velocity to RV peak A-wave KEi_EDV_		0.58	<0.01
E/a ratio to KEi_EDV_ E/A ratio		0.62	<0.01

RV = right ventricle, RVEDVi = right ventricular end-diastolic volume indexed, RVESVi = right ventricular end-systolic volume indexed, RVSVi = right ventricular volume indexed, RVEF = right ventricular ejection fraction, RAAi = right atrial area indexed, TAPSE = tricuspid annular plane systolic excursion, TV SV = tricuspid valve stroke volume, KEiEDV = kinetic energy indexed to end-diastolic volume.

### Age-associated tricuspid flow changes

The early diastolic tricuspid inflow peak velocity (E-wave) did not demonstrate any association to age. However, the late diastolic tricuspid inflow peak velocity (A-wave), increased significantly with age (R = 0.4, P < 0.01) (Table 2). Both, TV inflow E/A ratio and TV SV were negatively associated with increased age (P < 0.01).

### Age-associated RV intra-cavity blood flow KE changes

**Figure 2:**
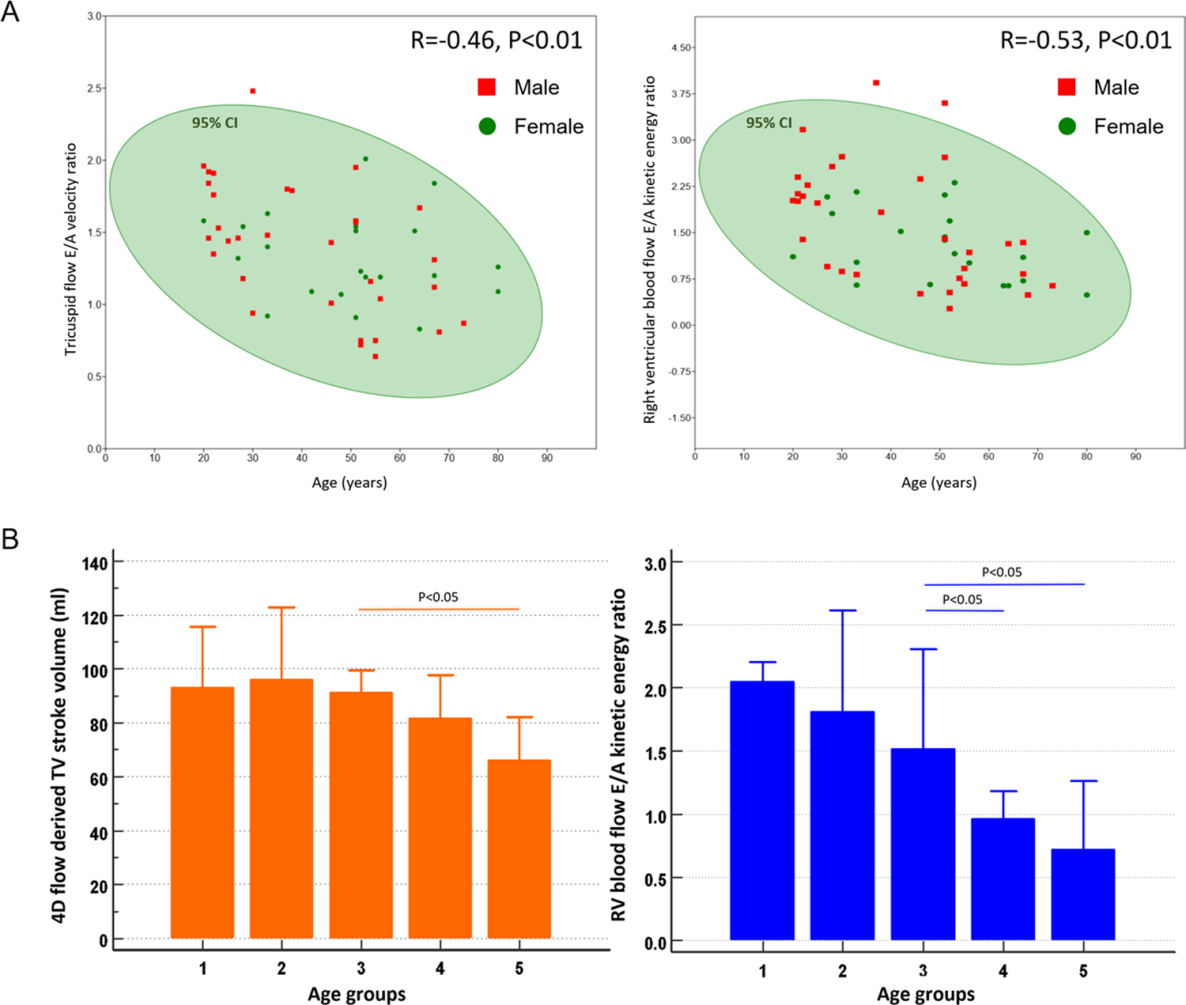
Panel A - scatter plots displaying the significant negative correlation between tricuspid flow E/A velocity ratio and age / right ventricular blood flow kinetic energy ratio and age. Panel B - Bar chart to display the values for 4D flow derived TV stroke volume and right ventricular blood flow E/A kinetic energy ratio. The x-axis denotes an increase in age, and the study population is divided into 5 groups.

RV global, systolic and diastolic KEi_EDV_ parameters demonstrated no significant changes with advancing age. However, peak E-wave KEi_EDV_ decreased with age whereas peak A-wave KEi_EDV_ increased with age. The RV KEi_EDV_ E/A ratio demonstrated the highest association to age (R = −0.53, P < 0.01) when compared to all RV functional and haemodynamic parameters (Fig. 2). All 2D tricuspid flow parameters were associated with their respective RV KE parameters (P < 0.01). The TV SV positively correlated with RV KEi_EDV_ E/A ratio (R = 0.36, P < 0.01).

### Regression analysis

In univariate analyse, indexed RV end-diastolic volume, TAPSE, RV peak A-wave velocity, E/A ratio, TV SV, RV peak E-wave KEi_EDV_, RV peak A-wave KEi_EDV_ and KEi_EDV_ E/A ratio demonstrated association to age (Table 3). In a multivariate model, only 4D flow derived TV SV and RV KEi_EDV_ E/A demonstrated independent association to age.

**Table 3:** Results of univariate and multivariate linear regression analysis of 2D velocity and 4D kinetic energy parameters.

Variables	Univariate		Multivariate	
	Coefficient β (SE)	P-value	Coefficient β (SE)	P-value
RVEDVi	−6.15 (2.04)	<0.01		
TAPSE	−1.57 (0.58)	<0.01		
RV peak A-wave velocity	2.66 (0.98)	<0.01		
E/A ratio	−0.12 (0.03)	<0.01		
TV SV	−7.59 (1.95)	<0.01	−0.02 (0.007)	<0.01
RV peak E-wave KEi_EDV_	−0.57 (0.26)	0.031		
RV peak A-wave KEi_EDV_	0.49 (0.17)	<0.01		
KEi_EDV_ E/A ratio	−0.29 (0.07)	<0.01	−0.68 (0.21)	<0.01

RV = right ventricle RVEDVi = right ventricular end diastolic volume indexed, TAPSE = tricuspid annular plane systolic excursion, TV SV = tricuspid valve stroke volume.

### Age group variations

**Figure 3:**
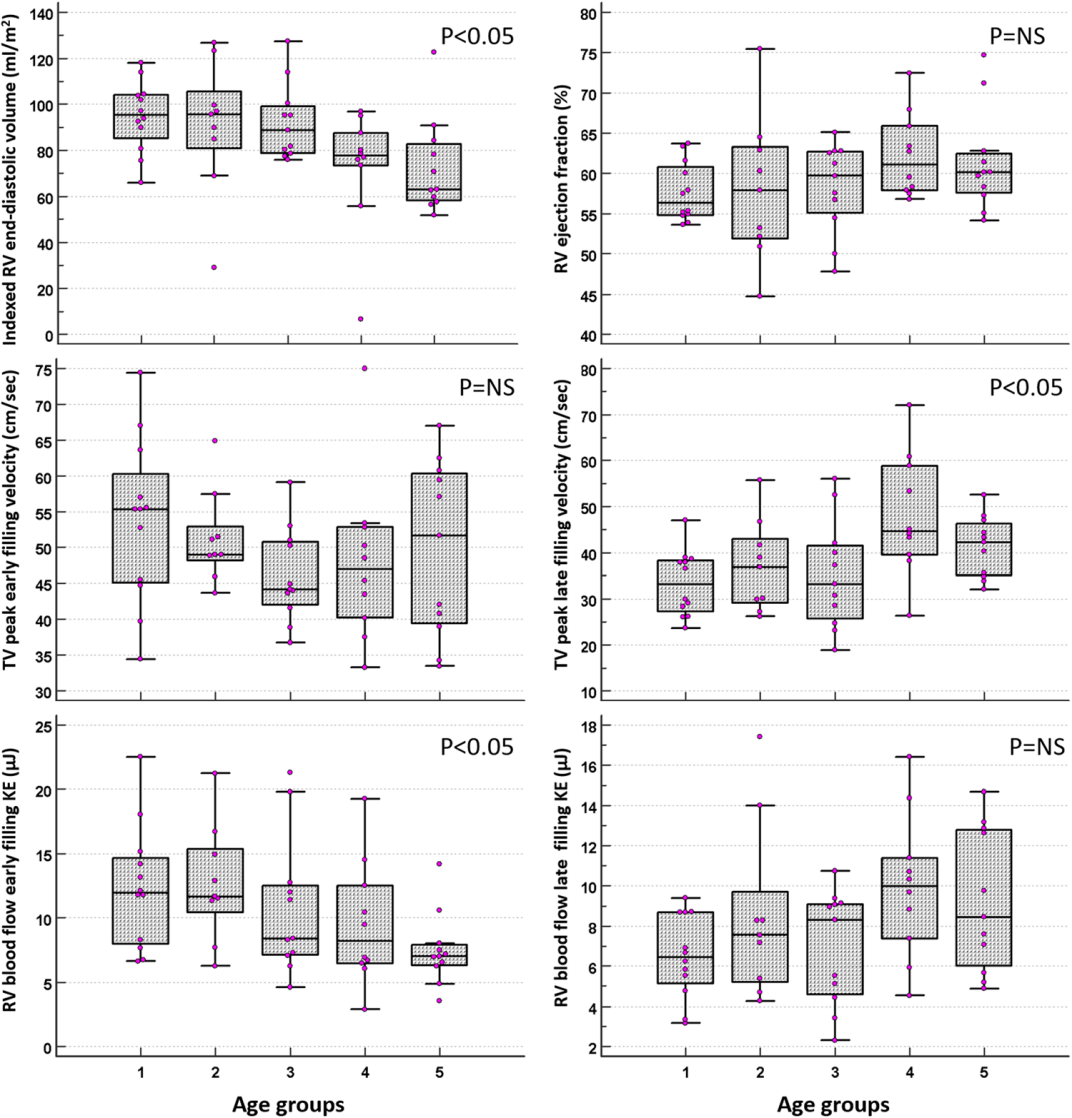
Box and whisker diagrams to demonstrate the association between age and right ventricular blood flow parameters. RV end-diastolic volume was found to have a significant negative association with age across the 5 age groups. The same was found for RV blood flow early filling KE. TV peak late filling velocity has a significant positive correlation with age across the 5 age groups. The rest of the parameters were found to be none significant when increasing with age.

The healthy volunteers were split into 5 groups according to age. Group 1 (n = 12) 23 ± 2 years old, group 2 (n = 9) 32 ± 3, group 3 (n = 11) 47 ± 4, group 4 (n = 10) 54 ± 2, group 5 (n = 11) 69 ± 6 years old (Table 4). On post-hoc analysis for inter age group changes, RVEDVi, RVESVi, RVSVi, peak A-wave TV flow velocity, TV E/A ratio, 4D flow derived TV SV and RV KEi_EDV_ E/A ratio demonstrated significant changes (Figs. 2, 3).

**Table 4:** Post-hoc analysis, divided according to age groups. Analysis of, both indexed and non-indexed for end-diastolic volume, volunteer haemodynamic and KE variables. The data is presented as the median ± interquartile range. The superscript letters (a–e) represent statistically significant inter-age group comparisons where P < 0.05.

Age groups	(a) (n = 12)	(b) (n = 9)	(c) (n = 11)	(d) (n = 10)	(e) (n = 11)	P
Mean age (yrs)	23 ± 2	32 ± 3	47 ± 4	54 ± 2	69 ± 6	
RVEDVi (ml/m^2^)	95.4 ± 19^d,e^	95.8 ± 24.6^d,e^	88.9 ± 20.3^d,e^	77.7 ± 14.1^a,b,c^	62.9 ± 24.6^a,b,c^	0.01
RVESVi (ml/m^2^)	39.3 ± 12.2^d,e^	36.4 ± 20.1^e^	34 ± 12.7^e^	31.8 ± 11.9^a^	26.1 ± 10.8^a,b,c^	0.01
RVSVi (ml/m^2^)	57 ± 8.18^d,e^	55.2 ± 7.3^e^	51.5 ± 12.2	48.2 ± 6.4^a^	39 ± 19.3^a,b^	0.04
RV EF (%)	56.4 ± 6	57.9 ± 11.4	59.7 ± 7.7	61.1 ± 8	60.1 ± 4.9	0.35
RAAi (mm)	11.6 ± 2.4	12.9 ± 3.3	13 ± 3	11 ± 1.4	12.7 ± 3.2	0.26
TV peak E-wave velocity (cm/s)	55.4 ± 14.9	49 ± 4.8	44.2 ± 8.7	47 ± 12.7	51.7 ± 21	0.34
TV Peak A-wave velocity (cm/s)	33.2 ± 11^d,e^	36.9 ± 13.7^d^	33.3 ± 15.9^d^	44.6 ± 19.3^a,b,c^	42.3 ± 11.3^a^	0.02
E/A ratio	1.6 ± 0.4^d,e^	1.5 ± 0.6^d^	1.5 ± 0.5^d^	1.1 ± 0.4^a,b,c^	1.2 ± 0.5^a^	0.01
TV SV (ml)	93.1 ± 38.9^e^	96.3 ± 39.2^e^	91.6 ± 20.5^e^	81.7 ± 36	66.3 ± 27.5^a,b,c^	0.02
TAPSE	29 ± 8.5	29.9 ± 9.3	29.6 ± 13.1	26.8 ± 6.9	25.6 ± 8.9	0.2
RV global KEi_EDV_ (µJ/ml)	4.3 ± 1.5	4.1 ± 2.3	4.9 ± 2.6	4.5 ± 1	3.8 ± 2	0.62
RV systolic KEi_EDV_ (µJ/ml)	8 ± 3.5	5.6 ± 5.6	8.3 ± 3.8	7.5 ± 3.4	6.8 ± 3.4	0.6
RV diastolic KEi_EDV_ (µJ/ml)	2.6 ± 1	2.9 ± 1.1	2.3 ± 1.2	2.3 ± 0.9	1.8 ± 1.2	0.36
RV peak E-wave KEi_EDV_ (µJ/ml)	5.6 ± 4	6.1 ± 3.7	3.7 ± 3.4	4.7 ± 3.7	4 ± 1.2	0.09
RV peak A-wave KEi_EDV_ (µJ/ml)	3.5 ± 1.8	3.9 ± 2.9	4.3 ± 2.2	5.1 ± 1.6	4.8 ± 4.2	0.1
RV KEi_EDV_ E/A ratio	2.1 ± 0.3^d,e^	1.8 ± 1.8^d.e^	1.5 ± 1^d,e^	1 ± 0.5^a,b,c^	0.7 ± 0.7^a,b,c^	<0.01
RV global KE (mJ)	8.6 ± 2.3	8.2 ± 3.5	8 ± 4.4	8.7 ± 3.8	6.1 ± 3.9	0.15
RV systolic KE (mJ)	16 ± 4	12.9 ± 8.5	14.1 ± 5.5	15.2 ± 8.1	10.3 ± 5.4	0.23
RV diastolic KE (mJ)	5.2 ± 1.6	5.3 ± 2.5	4.3 ± 2.5	4.4 ± 1.5	3.3 ± 1.6	0.09
RV peak E-wave KE (mJ)	12 ± 6.7^e^	11.7 ± 5^e^	8.4 ± 5.4	8.2 ± 6	7 ± 1.6^a,b^	0.05
RV peak A-wave KE (mJ)	6.5 ± 3.5	7.6 ± 4.5	8.3 ± 4.5	10 ± 4	8.5 ± 6.8	0.13

RV = right ventricle, RVEDVi = right ventricular end-diastolic volume indexed, RVESVi = right ventricular end-systolic volume indexed, RV SVi = right ventricular stroke volume indexed, TV SV = tricuspid valve stroke volume TAPSE = tricuspid annular plane systolic excursion, KEiEDV = kinetic energy indexed to end-diastolic volume.

### Intraobserver and interobserver reliability tests

Detailed reproducibility tests are in the online supplementary material. All of the indexed global KE parameters for the intraobserver reliability tests produced excellent correlation concordance coefficient (CCC) (average CCC 0.95, average precision 0.95, and average accuracy 0.99). The mean bias was also not significant for intraobserver tests (bias = −7%, P = NS). Overall, the interobserver CCC was acceptable at 0.90, average precision 0.9, and average accuracy 0.98) excluding peak E-wave KE. The interobserver peak E-Wave KE CCC was low at 0.59. The mean bias for interobserver tests was also not statistically different (bias − 6%, P = NS).

## Discussion

**Figure 4:**
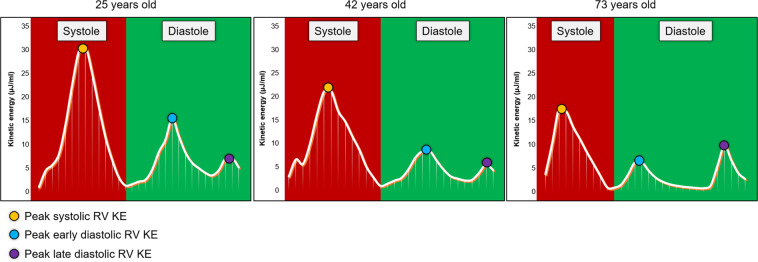
Three line graphs, in participants aged 25, 42 and 73 years old, to demonstrate the three blood flow kinetic energy peaks during the cardiac cycle during diastole and systole. The yellow dot shows the systolic peak of the kinetic energy curve. The blue dot denotes the E-wave peak of the kinetic energy curve. The purple dot is on top of the A-wave peak of the kinetic energy curve. The systolic peak decreases as age increases. Regarding diastole, early tricuspid inflow blood flow kinetic energy sharply decreases, and there is no significant pattern to late tricuspid filling. At age 73 it can be seen that the time for diastole is much longer, this is a compensatory mechanism to allow more time for the ventricle to fill as the kinetic energy of the blood flow is not very high. The x-axis gives the time through the cardiac cycle. The y-axis shows the kinetic energy, in µJ/ml.

In this study, we have shown that retrospective valve tracking derived tricuspid valve stroke volume and RV intra-cavity blood flow KEi_EDV_ E/A ratio are independently associated with ageing. This study also demonstrates that with healthy ageing, RV peak E-wave KEi_EDV_ decreases whereas RV A-wave KEi_EDV_ goes up (Fig. 4). In addition, this study demonstrates the reproducibility of the RV intracavity blood flow KE quantification methods.

In this study, the RV blood flow KE mapping was performed by a semi-automated method following the segmentation of RV in the short-axis cines. One of the key advantages of this is substantive time-saving. This is because no additional segmentation is required to derive KE parameters, and it takes less than a minute when compared to the automated retrospective valve tracking method, which takes around 4 minutes per-valve^27^. Moreover, in this study, RV diastolic KE parameters demonstrated good correlation to similarly defined 2D tricuspid flow metrics derived by retrospective valve tracking method. This becomes even more relevant as RV blood flow KE E/A ratio demonstrated superior association to ageing than any other RV parameter and hence may represent a better imaging marker of RV diastolic assessment, once age-adjusted normal RV blood flow KE E/A ratio tables are established enabling one to detect diastolic dysfunction of the right ventricle.

The reported individual components of RV blood flow KE in this study are similar to previously published studies^28,29^. Carlsson *et al*. demonstrated that the systolic peak of RV KE is larger than the early diastolic peak. This study also confirms these universal physiological findings. Fredriksson *et al*. demonstrated that the majority of RV KE comes from direct flow, blood that enters the ventricle during diastole and leaves the ventricle during systole in the analysed heartbeat. The results from our study demonstrated that the RV global KE for the complete cardiac cycle was comparable in the different age groups that we studied (P > 0.05) and had no association to age, within the limitation of the cohort we studied. A plausible explanation for this is that the global RV KE for the complete cardiac cycle is preserved throughout healthy ageing and only individual components may change. We do not have data on older healthy adults to ascertain that this finding is universally applicable throughout the ageing process. Even RV blood flow systolic and diastolic average KEi_EDV_ did not show any significant association to ageing. However, peak blood flow KE during early and late ventricular filling demonstrated association to ageing and their ratio significantly decreased with ageing. These findings are very similar to LV blood flow KE and suggest that RV diastolic filling also adapts in a similar way to LV filling and the two share a common physiology. One explanation for our finding is that as RV diastolic function deteriorates with ageing due to stiffness of the RV^17^, there is a steady decrease in E-wave KE with a compensatory increase in RV peak A-wave KEi_EDV.,_ These results support the previously established theory that an increase in right atrial contraction and its associated rise in right atrial pressure are required to maintain sufficient right ventricular filling in older individuals^30^.

Previous studies using echocardiography have similarly shown that with advancing age there is a reduction in the early filling velocity of the RV^17,30^. Lindqvist *et al*. used pulse wave Doppler imaging to determine RV early and late filling velocities and similar to our work, their study demonstrated that peak E-wave velocity of tricuspid inflow decreases with advancing age and peak A-wave velocity increases with increased age^30^. In both this previous and our current study there was a consistent significant increase in tricuspid peak A-wave diastolic velocities (r = 0.35, P < 0.01 vs ours, r = 0.4, P < 0.01). Additionally, the E/A ratio declined significantly as age increased in both studies (Lindqvist, r = −0.57, P < 0.01 vs ours, r = −0.46, P < 0.01). However, no reliability tests were carried out for Doppler methods. Doppler methods are subject to through-plane motion which can significantly alter the results^31^. In another large study which included 298 healthy subjects, peak early filling tricuspid flow velocity ratio to early diastolic tissue Doppler velocity (Ea) demonstrated only a modest correlation to ageing (r = 0.21, P < 0.01) versus the 4D flow derived RV blood flow KE E/A ratio which demonstrated much better correlation to ageing (r = −53, <0.01). As age-dependent changes of RV diastolic properties can be attributed to the increase of the arterial stiffness of the pulmonary vessels occurring with ageing, however, more work is required to determine that the RV blood flow KE E/A ratio can be a better imaging marker of raised pulmonary artery pressures^32,33^.

As per previous literature by Fiechter *et al*., the end-diastolic volume, end-systolic volume and RV stroke volume in our study also had a significant negative correlation with age (P < 0.01)^9^. All of these results support the current literature that ageing is associated with a number of physiological RV volumetric and flow changes^3,4^.

For the clinical translation of any novel imaging marker, other than diagnostic accuracy, good intra-/interobserver reproducibility are very important. In this study, all the RV blood flow KE parameters demonstrated a high degree of reproducibility, in particular, the RV blood flow KE E/A ratio had only 1.4% and 3.5% intra-inter-observer bias respectively with acceptable CCC for clinical translation.

It is worth mentioning that in our study there were no major gender-related differences both for RV volumetric and blood flow assessment. This is probably because the number of healthy individuals recruited in this study is small as opposed to other population-based CMR studies which have previously demonstrated that males tend to have larger RV volume^34^. Future studies are warranted to investigate RV blood flow differences in a larger CMR study.

### Clinical perspective

Lindqvist *et al*. have previously elicited that age does not impact RV systolic function, the changes in RV function, with age, are mainly due to diastolic filling mechanisms. Thus, in clinical practice, there is a need for accurate and reliable quantification of RV diastolic function. Current methods to assess RV diastolic function have not been translated into clinical practice due to their limitations. This study paves the way for novel semi-automated strategies to construct a comprehensive RV diastolic function assessment with a high degree of intra-/inter-observer reproducibility for the majority of the metrics. It provides a reliable parameters’ correlation with age that could produce once expanded into an older adult, a comprehensive table of the normal age-adjusted table against which comparisons could be made when studying patients with possible right ventricular impairment. Further studies are warranted to investigate and develop non-invasive models using the methods proposed against invasively derived right heart pressures for their clinical translation.

### Limitations

During the 4D flow acquisition, respiratory navigation was omitted which may have had an impact on RV KE parameters. However, studies that carried out a head-to-head comparison of whole-heart 4D flow have demonstrated that for quantification of intra-cardiac KE, both respiratory navigated and non-respiratory navigated 4D flow acquisitions are comparable^35^. Another limitation that could influence the quality of the KE data, is a low temporal resolution (40 ms). Spatial co-registration of breath-hold cine and free-breathing 4D flow to derive KE could have introduced some quantification errors. Other confounding factors include variation in the heart rate and physiological condition between the two acquisitions. Additionally, the data gathered from this study cannot be used for patients that suffer from congenital heart disease, valvular disease and or cardiomyopathies. This study was done in healthy volunteers and we did not record any lifestyle risk factors in these volunteers.

Finally, we believe that extending the study to include older healthy volunteers is necessary before we could propose a full age-adjusted normal table of measurement of RV blood flow KE E/A ratio, against which patients could be compared.

## Supplementary information

Supplementary Information.
