# Supplementary material for: Age-associated changes in 4D flow CMR derived Tricuspid Valvular Flow and Right Ventricular Blood Flow Kinetic Energy
Source: Sci Rep. 2020 Jun 18;10:9908. doi: 10.1038/s41598-020-66958-y (PMC7303161; doi:10.1038/s41598-020-66958-y)
Supplement: Supplementary file 1 — Supplementary Information. [file 41598_2020_66958_MOESM1_ESM.docx]

Age-associated changes in 4D flow CMR derived Tricuspid Valvular Flow and Right Ventricular Blood Flow Kinetic Energy

Type of Article: Research Article

*Natasha Barker^1^, *Hamza Zafar^1^, Benjamin Fidock^1^, Alaa Elhawaz^1^, Abdallah Al-Mohammad^1,2^, Alexander Rothman^1^, David G. Kiely^1,2,3^, R.J. van der Geest^4^, Jos Westenberg^4^, Andrew J. Swift^1,3^, James M. Wild^1,3^, Sven Plein^5^, Pankaj Garg^1^

^*^Shared first co-authorship as equal contribution

^1.^ Department of Infection, Immunity & Cardiovascular Disease, University of Sheffield, Sheffield, UK

^2.^ Sheffield Teaching Hospitals NHS Foundation Trust, Sheffield, UK

^3.^ Insigneo Institute for in-silico Medicine, Sheffield

^4.^ Cardiovascular Imaging Group, Department of Radiology, Leiden University Medical Centre

^5.^ Division of Biomedical Imaging, Leeds Institute of Cardiovascular and Metabolic Medicine, University of Leeds, Leeds, UK

#

Corresponding Author:

Dr Pankaj Garg MD (Hons), PhD

Clinical Lecturer in Cardiovascular Medicine

Department of Infection, Immunity & Cardiovascular Disease

University of Sheffield

Medical School

Sheffield S10 2RX

ORCID ID: 0000-0002-5483-169X

E: p.garg@sheffield.ac.uk

T: +44 (0)114 215 9152

# Supplementary online document

1. Defining right ventricular endocardial contours
2. Supplementary Table 1. Detailed intraobserver and interobserver results

Defining right ventricular endocardial contours

The septal boundary of the right ventricular cavity was defined by the left ventricular epicardial contour. The right ventricular endocardial contour was drawn on top of the left ventricular epicardial contour in this region. However, in the most basal slices, where there was no visible septal wall, the right ventricular endocardial contour was not connected to the left ventricular epicardial contour.

The papillary muscles and trabeculations were kept within the defined endocardial contours. The cine view and movie mode were helpful in identifying how these structures move and deform over the cardiac cycle.

Basal RV contouring remains challenging. Hence, we followed a rigid protocol to make sure the RV was contoured in exactly the same way between two-operators. For all the basal slice the contours were drawn manually, as the separation between the right ventricle and right atrium blood pool was often poorly defined. Only the volumes below the pulmonary valve were contoured. If the pulmonary valve was visible, the region below it was contoured

Further decisions for which phase to include or exclude in the basal slices was made using the four-chamber view (4CH) (Supp. Fig 1 and 2). The 4CH view was used to assess the position of the tricuspid annulus plane in that phase of the cardiac cycle.

Finally, the volume curves were used to check there were no sudden spikes or dips, and therefore the correct area was being traced. The right ventricular contours in the basal slice gradually reduced in size as they got closer to the pulmonary valve, shown in Supp. Fig 1 and 2. This was done to avoid large changes in contour area from one phase to the next, to allow for fluidity of the contours through the phases and a smooth volume curve. These volume curves were generated to obtain relevant cardiac physiological parameters, including right ventricular end-diastolic volume, right ventricular end-systolic volume, right ventricular stroke volume and right ventricular ejection fraction.

Supplementary Figure 1. Most basal slice: RV segmentation avoided if the slice is in the right atrium on 4-chamber view.

**
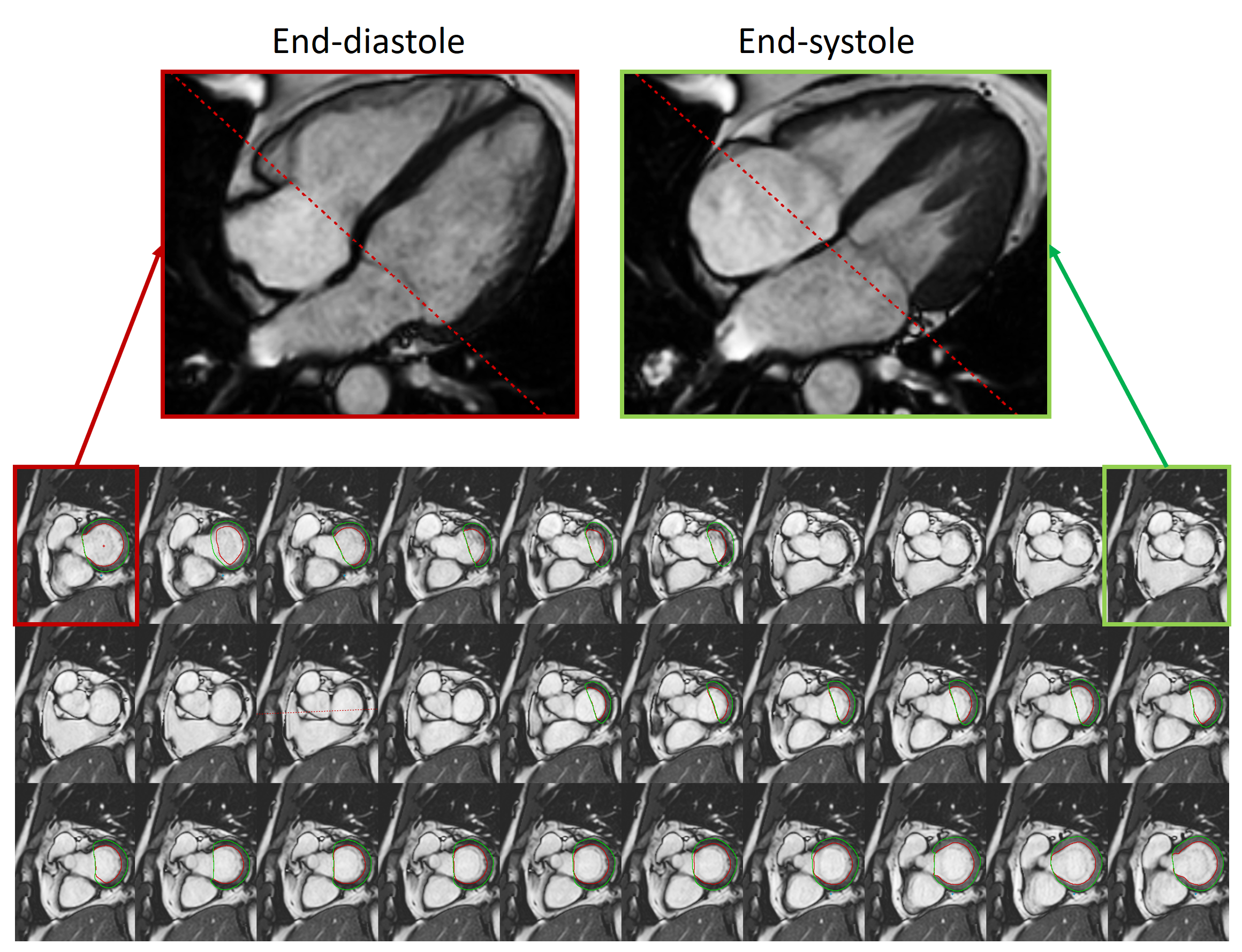
**

Supplementary Figure 2. Basal slice of the right ventricle. This is the most basal slice where RV was contoured but not for all cardiac phases. The end-diastolic phase has a red border, the end-systolic phase has a green border. The mid-systolic phase where the RV came out of the view due to through-plane motion, is the orange-bordered image. The reappearance of the RV during diastole is shown with blue borders.

**
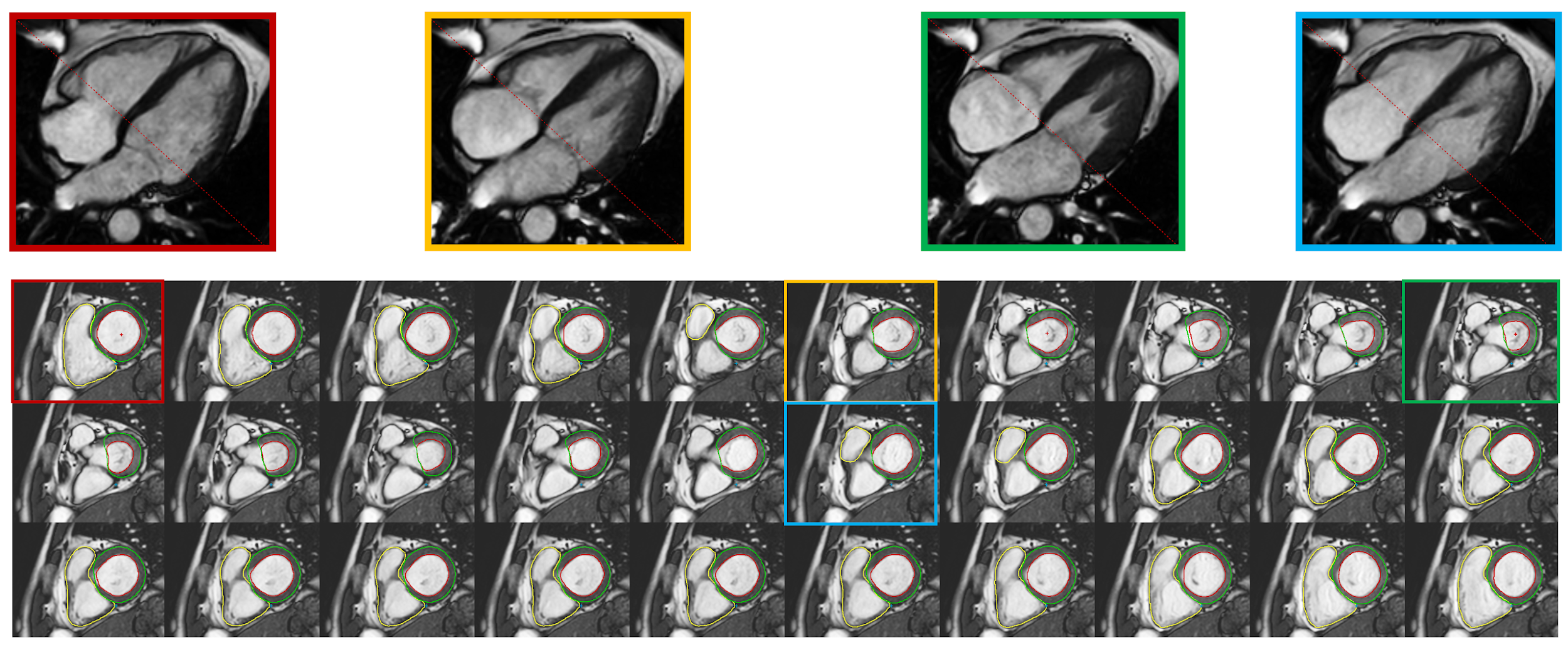
**

Supplementary Table 1. Detailed intraobserver and interobserver results for all the RV intra-cavity blood flow KE parameters.

| **Intra-observer tests** | | | | |
| --- | --- | --- | --- | --- |
|  | **Bias (95% CI)** | **UL (95% CI)** | **LL (95% CI)** | **P-value** |
| RV global KEi_EDV_ | -7.7 (-15.9 to 0.5) | 26.6 (12.4 to 40.8) | -42 (-56.3 to -27.8) | 0.06 |
| RV systolic KEi_EDV_ | -3.3 (-10.2 to 3.6) | 25.6 (13.6 to 37.6) | -32.2 (-44.2 to -20.2) | 0.32 |
| RV diastolic KEi_EDV_ | -9 (23.5 to 5.3) | 51.1 (26.1 to 76.1) | -69.3 (-94.3 to -44.3) | 0.20 |
| RV peak E-wave KEi_EDV_ | -25.2 (-76 to 25.6) | 187.6 (99.2 to 275.9) | -238 (-326.4 to -149.7) | 0.31 |
| RV A-wave KEi_EDV_ | 1.8 (-3.9 to 7.6) | 26 (15.9 to 36) | -22.3 (-32.3 to -12.3) | 0.52 |
| KEi_EDV_ E/A ratio | -1.4 (-6.7 to 3.7) | 20.8 (11.5 to 30) | -23.6 (-32.8 to -14.4) | 0.58 |
|  | **CCC (95% CI)** | **Pearson ρ (precision)** | **Cb (accuracy)** | **P-value*** |
| RV global KEi_EDV_ | 0.96 (0.91 to 0.98) | 0.97 | 0.99 | <0.01 |
| RV systolic KEi_EDV_ | 0.97 (0.94 to 0.99) | 0.98 | 0.99 | <0.01 |
| RV diastolic KEi_EDV_ | 0.91 (0.78 to 0.96) | 0.91 | 0.99 | <0.01 |
| RV peak E-wave KEi_EDV_ | 0.91 (0.79 to 0.96) | 0.91 | 0.99 | <0.01 |
| RV A-wave KEi_EDV_ | 0.95 (0.88 to 0.98) | 0.95 | 0.99 | <0.01 |
| KEi_EDV_ E/A ratio | 0.98 (0.95 to 0.99) | 0.98 | 0.99 | <0.01 |
| **Inter-observer tests** | | | | |
|  | **Bias (95% CI)** | **UL (95% CI)** | **LL (95% CI)** | **P-value** |
| RV global KEi_EDV_ | -0.4 (-8.4 to 9.3) | 37.5 (22.1 to 52.9) | -36.7 (-52.1 to -21.3) | 0.92 |
| RV systolic KEi_EDV_ | 3.8 (-8.5 to 16.1) | 55.2 (33.9 to 76.6) | -47.62 (-69 to -26.3) | 0.53 |
| RV diastolic KEi_EDV_ | -1.3 (-7.2 to 4.7) | 23.6 (13.3 to 33.9) | -26.1 (-36.4 to -15.8) | 0.66 |
| RV peak E-wave KEi_EDV_ | -33.3 (-98.9 to 32.3) | 241.4 (127 to 355) | -308 (-422 to -194) | 0.30 |
| RV A-wave KEi_EDV_ | -0.6 (-6.4 to 5.2) | 23.7 (13.6 to 33.8) | -25 (-35 to -14.9) | 0.82 |
| KEi_EDV_ E/A ratio | -3.5 (-20.7 to 13.6) | 68.3 (38.5 to 98.1) | -75.3 (-105.2 to -45.5) | 0.67 |
|  | **CCC (95% CI)** | **Pearson ρ (precision)** | **Cb (accuracy)** | **P-value*** |
| Rv global KEi_EDV_ | 0.93 (0.83 to 0.97) | 0.93 | 0.99 | <0.01 |
| RV systolic KEi_EDV_ | 0.82 (0.61 to 0.92) | 0.84 | 0.97 | <0.01 |
| RV diastolic KEi_EDV_ | 0.95 (0.88 to 0.98) | 0.95 | 1.00 | <0.01 |
| RV peak E-wave KEi_EDV_ | 0.59 (0.25 to 0.8) | 0.62 | 0.94 | <0.01 |
| RV A-wave KEi_EDV_ | 0.95 (0.88 to 0.98) | 0.95 | 0.99 | <0.01 |
| KEi_EDV_ E/A ratio | 0.82 (0.61 to 0.92) | 0.83 | 0.99 | <0.01 |
| *The p-value is derived using is Pearson correlation.CCC: concordance coefficient correlation, UL: upper limits of agreement, LL: lower limits of agreement, CI: confidence interval, RV: right ventricle, KE: kinetic energy. | | | | |
